# Does crossing the pond affect crystal quality?

**DOI:** 10.1063/4.0001122

**Published:** 2025-10-27

**Authors:** Christopher S. Campomizzi, M. Elizabeth Snell, Halina Mikolajek, James Sandy, Juan Sanchez-Weatherby, Gabrielle R. Budziszewski, Silvia Russi, Aina E. Cohen, Michael A. Hough, Sarah E. J. Bowman

**Affiliations:** 1 University at Buffalo - Hauptman Woodward Research Institute, Buffalo, NY, USA; 2 Department of Biochemistry, Jacobs School of Medicine and Biomedical Sciences, SUNY Buffalo, Buffalo, NY, USA; 3 Diamond Light Source Ltd, Harwell Science and Innovation Campus, Didcot, England, United Kingdom; 4 The Research Complex at Harwell, Harwell Science and Innovation Campus, Didcot, England, United Kingdom; 5 Stanford Synchrotron Radiation Lightsource, SLAC National Accelerator Laboratory, Stanford University, Menlo Park, CA, USA

## Abstract

Room-temperature (RT) X-ray diffraction experiments can be carried out in situ, directly on crystallization trays without any manipulation of protein crystals, improving crystal integrity for fragile crystals. Further, RT experiments enable us to investigate protein dynamics, efficiently probe fragment binding, and perform time-resolved crystallography experiments. The Versatile Macromolecular Crystallography in-situ (VMXi) beamline at Diamond Light Source (DLS) in the United Kingdom specializes in the collection of RT X-ray diffraction data directly from in-situ crystallization trays. While many X-ray sources are now equipped to grow crystals on site for in-situ experiments, to date there has been no comprehensive analysis that we are aware of on the effect of shipping crystals in plates at ambient temperature for RT data collection. Here we examine the impact of shipping on crystals grown on MiTeGen In-Situ-1 plates at the University of Buffalo Hauptman Woodward Research Institute (UB-HWI) in Buffalo, NY. The in-situ crystals were shipped transatlantic in Blue Boxes designed at Stanford Synchrotron Radiation Lightsource (SSRL) to DLS in the UK. We hypothesized that long-distance shipping might compromise data quality through mechanical stress or temperature fluctuations. Surprisingly, room-temperature data collected at VMXi showed no significant differences for crystals set up at UB-HWI and shipped relative to crystals set up on-site at DLS. High-resolution structures were successfully determined for all proteins in the study, demonstrating that long-distance shipment of crystals at non-cryogenic temperatures is feasible without compromising diffraction quality. This study provides a proof- of-concept workflow for expanding access to room temperature crystallography worldwide, enabling more researchers to leverage cutting-edge techniques without needing to grow crystals on site.

